# GIBBSTHUR: Software for Estimating Variance Components and Predicting Breeding Values for Ranking Traits Based on a Thurstonian Model

**DOI:** 10.3390/ani10061001

**Published:** 2020-06-08

**Authors:** Luis Varona, Andrés Legarra

**Affiliations:** 1 Departamento de Anatomía, Embriología y Genética Animal, Instituto Agroalimentario de Aragón (IA2), Universidad de Zaragoza, 50018 Zaragoza, Spain; 2 Génétique Physiologie et Systèmes d’Elevage (GenPhySE), Institut National de la Recherche Agronomique de Toulouse, 31326 Castanet-Tolosan, France; andres.legarra@inrae.fr

**Keywords:** ranking traits, horse, breeding, Thurstonian model, Gibbs sampler

## Abstract

**Simple Summary:**

This article describes a new software (GIBBSTHUR) that provides Bayesian estimation of variance components and predictions of breeding values for ranking traits generated from equine competitions based on a Thurstonian approach. The GIBBSTHUR software was developed in FORTRAN 90 and can be executed in UNIX, OSX, or WINDOWS environments, and is freely available in a public repository (https://github.com/lvaronaunizar/Gibbsthur).

**Abstract:**

(1) Background: Ranking traits are used commonly for breeding purposes in several equine populations; however, implementation is complex, because the position of a horse in a competition event is discontinuous and is influenced by the performance of its competitors. One approach to overcoming these limitations is to assume an underlying Gaussian liability that represents a horse’s performance and dictates the observed classification in a competition event. That approach can be implemented using Montecarlo Markov Chain (McMC) techniques with a procedure known as the Thurstonian model. (2) Methods: We have developed software (GIBBSTHUR) that analyses ranking traits along with other continuous or threshold traits. The software implements a Gibbs Sampler scheme with a data-augmentation step for the liability of the ranking traits and provides estimates of the variance and covariance components and predictions of the breeding values and the average performance of the competitors in competition events. (3) Results: The results of a simple example are presented, in which it is shown that the procedure can recover the simulated variance and covariance components. In addition, the correlation between the simulated and predicted breeding values and between the estimates of the event effects and the average additive genetic effect of the competitors demonstrates the ability of the software to produce useful predictions for breeding purposes. (4) Conclusions: the GIBBSTHUR software provides a useful tool for the breeding evaluation of ranking traits in horses and is freely available in a public repository (https://github.com/lvaronaunizar/Gibbsthur).

## 1. Introduction

Ranking traits are used frequently as selection criteria in competitive horse breeding programs [1,2]; however, the position of a horse in a competitive event has a discontinuous nature and is influenced by the performance of its competitors, which makes genetic evaluations based on standard linear models difficult. Several transformations for overcoming that limitation have been proposed—e.g., the squared root of ranks [3], the Snell transformation score [4,5], and the normalized score [6].

Another interesting approach is to assume an underlying Gaussian liability that represents the horse’s performance and dictates the observed classification in competition events [1,7]. The original implementation of that approach involved a complex numerical integration [1], but Gianola and Simianer [8] proposed a Bayesian approach called the Thurstonian Model, which can be implemented using McMC methods such as the Gibbs Sampler [9]. In horse breeding, that model has been tested on simulated data [10] and has been applied to trotter horses [11] and the results of endurance competitions [12]. The practical use of the method has been limited, however, partly because of the absence of suitable and user-friendly software.

The objective of this note is to present a new free software (GIBBSTHUR) that implements the Bayesian estimation of variance components and the prediction of breeding values for ranking traits based on a Thurstonian Model. In addition, the software can analyze continuous and threshold traits jointly with the underlying liability of the ranking traits.

## 2. Theoretical Background

The Thurstonian Model assumes an underlying variable or liability (***l***) that is transformed into ranking in competitions (***t***). The model for the underlying variable can include systematic, random environmental and additive genetics effects, and a residual effect whose variance has to be predefined. Systematic effects generally include, among others, sex, year, or type of competition, but for ranking traits, it is interesting to include an “event” effect in order to take into account the heterogeneity of the level of the competitors linked with the prestige of the competitive events. The liability can be modelled alone or in conjunction with other continuous or threshold traits through the additive genetic, random environmental, or residual covariances between them.

To illustrate the model, suppose a simple bivariate model composed of ***l*** and a continuous Gaussian trait (***y***) which includes systematic and random additive genetic effects only. The probability distribution of ***l*** and ***y***, given the remaining parameters in the model, is the following multivariate Gaussian distribution (*MVN*):

$$\left(\begin{array}{c} l\\ y \end{array}\right)=MVN\left(\begin{array}{c} X_{l}b_{l}+Z_{l}a_{l}\\ X_{y}b_{y}+Z_{y}a_{y} \end{array},R⊗I\right),$$

where ***b_l_*** and ***b_y_*** and ***a_l_*** and ***a_y_*** are the systematic and additive genetic effects for the liability (***l***) and the continuous (***y***) traits, respectively. Furthermore, ***X_l_***, ***X_y_***, ***Z_l_***, and ***Z_y_*** are the corresponding incidence matrices, and ***R*** is the residual covariance matrix. The prior distribution of the additive genetic effects is the following multivariate Gaussian distribution:

$$\left(\begin{array}{c} a_{l}\\ a_{y} \end{array}\right)=MVN\left(\begin{array}{c} 0\\ 0 \end{array},G⊗A\right),$$

where ***A*** is the numerator relationship matrix and ***G*** is the additive genetic covariance matrix. In addition, the prior distributions of ***R*** and ***G*** are Inverted Wishart. The program uses a Gibbs Sampler approach [9] to obtain the posterior marginal estimates of all the unknowns in the model. The Gibbs sampler is an updating sampling scheme that requires sampling from the full conditional posterior distributions. The conditional distributions of systematic, random environmental, and additive genetic effects are univariate Gaussian, while the conditional distributions of the variance covariance matrices are Inverted Wishart. As with threshold traits, the Bayesian implementation of the model based on the Gibbs Sampler, after conditioning on liability, is similar to the bivariate model that has two continuous traits [13].

Therefore, the key step for the implementation within a McMC Bayesian approach is the defining of the conditional predictive distribution of the liabilities given the observed ranking data, as follows:

$$p\left(l_{kj}|ELSE\right)\propto \prod_{j=1}^{J}{\left[N\left(l_{kj}|\mu_{kj},\sigma_{kj}^{2}\right)\right]}^{\delta_{kl}}T_{k},$$

where *l_kj_* is the liability of the *j*th individual at the *k*th completion event and ELSE denotes the liabilities at the same and other competition events and the systematic and additive genetic effects for the ranking trait and the correlated Gaussian trait. Further,

$$\mu_{kj}=x_{lkj}b_{l}+z_{lkj}a_{l}-\left(y_{kj}-x_{ykj}b_{y}-z_{ykj}a_{y}\right)\times \frac{r^{12}}{r^{22}},$$

and

$$\sigma_{kj}^{2}=\frac{1}{r^{11}},$$

where $r^{ij}$ is the (*i*,*j*) element of $R^{-1}=\left(\begin{array}{cc} r^{11} & r^{12}\\ r^{12} & r^{22} \end{array}\right)$, and $x_{lkj}$, $x_{ykj}$, $z_{lkj}$, and $z_{ykj}$ are the corresponding rows of the $X_{l}$, $X_{y}$, $Z_{l}$, and $Z_{y}$ matrices that link the liability ($l_{kj})$ and the Gaussian ($y_{kj}$) trait for the *j*th data at the *k*th event with the vectors of systematic ($b_{l}$ and $b_{y}$) and additive genetic ($a_{l}$ and $a_{y}$) effects. In addition, $\delta_{kj}$ is 1 if the *j*th competitor is present at the *k*th event and 0 otherwise, and ***T_k_*** is an indicator set that denotes the order of liabilities within the *k*th competition. Therefore, the liabilities within each event are not independent, because the sample space is restricted by the ranking represented by ***T_k_*_._** The conditional sampling of liabilities is performed sequentially. First, the liability of the horse that finishes in position one is set to 0 to achieve the identifiability of the model, whereas an “event” effect is estimated. An alternative, equivalent option used in [10] is to fix the “event” effect to 0 and sample the first horse from a truncated normal conditional on the other competitors. Second, the liabilities of the remaining individuals in the competition event are calculated by sampling from the truncated Gaussian distributions (TN) that have a mean $\mu_{kj}$ and a variance $\sigma_{kj}^{2}$, with limits defined by the liabilities of the preceding horse and of the horse classified just after it.

## 3. The Software: Input and Output Files

The software (GIBBSTHUR) was developed in FORTRAN90 and is based on the TM software [14]. It is available at https://github.com/lvaronaunizar/Gibbsthur. Running the program requires a parameter file (‘parameters.txt’), which must include the names of the pedigree and data file; the number of additional traits and their nature (continuous, threshold, or ranking); the description of the model of analysis for all the traits (systematic, random, and genetic effects); and the hyperparameters of the Gibbs sampler implementation that comprise the total number of iterations, the number of burn-in iterations to discard, and the thin interval. The pedigree file must include a list of the individual, sire, and dam entries numbered from 1 to the number of individuals. The program allows the user to include genetic groups for unknown sires or dams. The data file must have an entry for each data, and should include the level for each systematic, random, and additive genetic effect, numbered from 1 to the maximum number of levels for each effect; and the performance for the continuous, threshold, and ranking traits. Missing data should be coded as 0. There is no limit to the number of competitors per competition. Empty positions are admitted in the results of competitions.

The execution of the program generates the following files: “Results.txt”, “Breeding.txt”, “Solutions.txt”, and “Iterations.txt”. The file “Results.txt” includes a summary of the posterior mean and standard deviations for the variance and covariance components; the heritabilities; the ratios of variance; and the additive genetic, permanent environmental, and residual correlations. They are computed with the output of the Gibbs sampler iterations after the burn-in. The file “Breeding.txt” includes the posterior mean and standard deviations of the breeding values for the ranking traits and the additional traits, and they can be directly used for selection purposes. The file “Solutions.txt” includes the posterior mean and standard deviations of the systematic and random environmental effects for the ranking and the additional traits. The file “Iterations.txt” includes the samples of each iteration of the Gibbs sampler for the variance and covariance components, which can be used to analyze in detail the marginal posterior distributions. A more detailed description is available at https://github.com/lvaronaunizar/Gibbsthur.

## 4. Simulation Example

**Figure 1:**
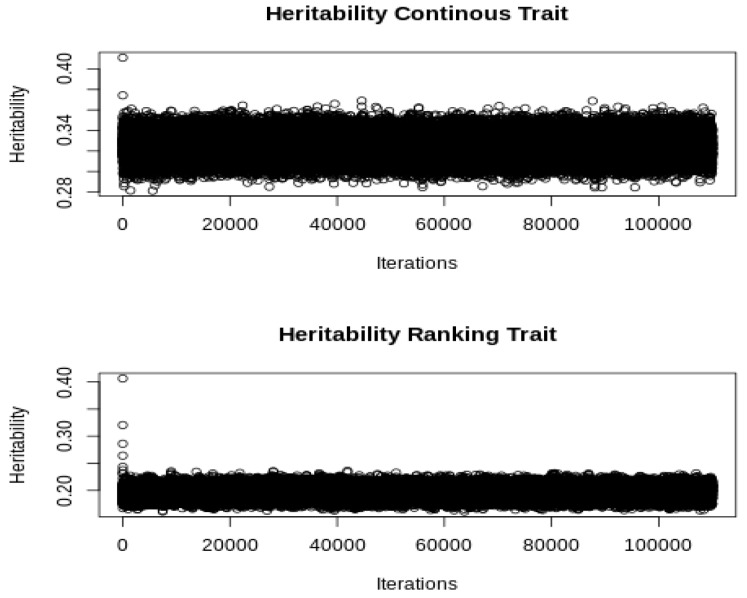
Plot of the Gibbs sampler iterations of the heritabilities of the continuous and ranking traits.

A simple pedigree comprising 100 sires and 500 dams generates 2000 horses that compete randomly in 2000 events of 10 horses each. On average, each horse competes in 10 events. The liability was generated by a model that included a general mean, one additive genetic effect, and a residual. The additive genetic and residual variances were set to 0.25 and 1 ($h_{l}^{2}$ = 0.2), respectively. In addition, we simulated a correlated continuous trait that had additive and residual variances of 1 and 2 ($h_{l}^{2}$ = 0.33), respectively, and additive genetic and residual correlations with the liability of the ranking trait of 0.3 and −0.2, respectively. The model of analysis with the GIBBSTHUR software included two systematic effects (a general mean and an event effect), and the additive genetic and residual random effects. We used a Gibbs sampler chain of 100,000 iterations after discarding the first 10,000. The initial values of variance components were established by dividing the raw phenotypic variance by the number of variance components in the model. The convergence was checked by a visual inspection (see Figure 1). The computing time was 51 min and 23 s with a i9-7960X processor (2.80 GHz).

## 5. Results and Discussion

**Figure 2:**
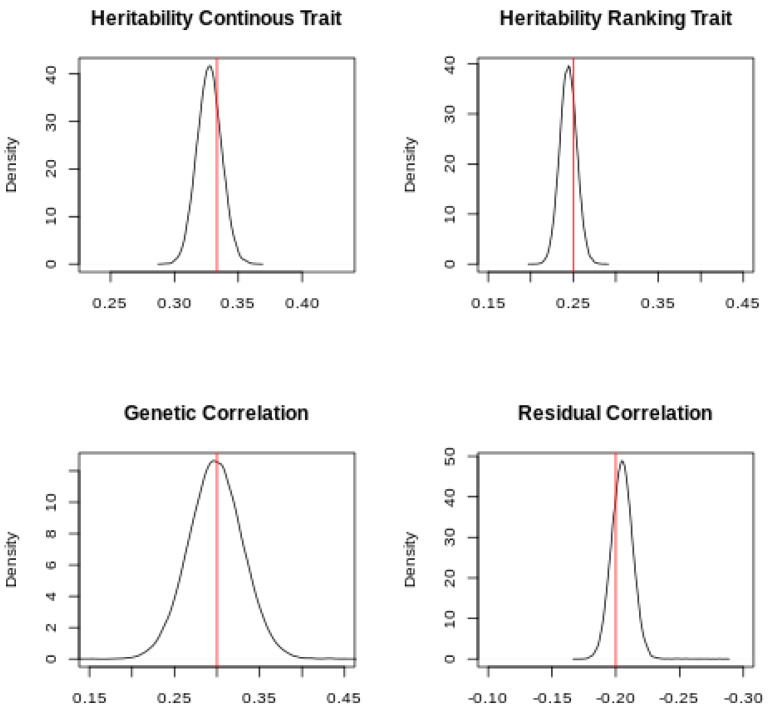
Posterior distribution and simulated parameters (in red) of the heritabilities of the continuous and ranking traits and of the genetic and residual correlations.

The posterior distribution of the heritabilities and the genetic and residual correlations is presented in Figure 2.

**Figure 3:**
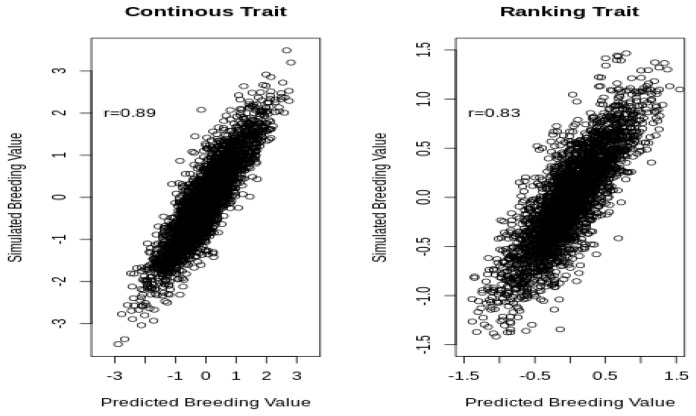
Two-dimensional plots of the simulated and predicted breeding values for the continuous and the liability of the ranking traits.

The simulated values were within the Highest Posterior Density (HPD) at 95% of the posterior distributions, which confirmed that the software can retrieve the simulated parameters for continuous and ranking traits. Slight differences between the mode of the posterior distributions and the simulated values can be expected, given the small size of the dataset. Figure 3 shows the predicted and simulated breeding values for the continuous and ranking traits.

The correlation between the predicted and the simulated breeding values was higher for the continuous (0.89) than it was for the liability of the ranking trait (0.83), although, in both cases, the correlation was very strong because the amount of information available for each horse was large (on average, 10 phenotypic records).

The ability to discriminate between the performances of the individuals in competition events is illustrated in Table 1, which provides a detailed description of the phenotypic information for the ranking traits of the best and worst predicted breeding values. The best individual won 8 of 13 races, and the horse with the worst breeding values came last in 5 of 8 races.

One of the main limitations of using ranking traits for the breeding evaluation of competitive horses is that competition events often are structured into categories based on their prestige or monetary reward and, therefore, the participation of horses in those events is not random, because the most prestigious events attract the best horses and the worst horses tend to participate in other events, which have a lower average performance. Gianola and Simianer [8] proposed including an “event” effect, although Legarra and Ricard [10] pointed out that “event” effects cannot be estimated from ranking data because the event does not have any influence on rankings, and they recommended removing them to achieve identifiability. Here, we used a different (but equivalent) approach that zeroed the liability of the best horse in each event, which forced the liabilities of the horses from the second to the last position to be negative. Therefore, the estimates of the event effects were also forced to be negative. In that approach, the systematic effect of the event is not an effect of the race per se (i.e., weather or track conditions), but is linked to the performance of the best horse and provides an indicator of the average level of the competitors in the event.

**Figure 4:**
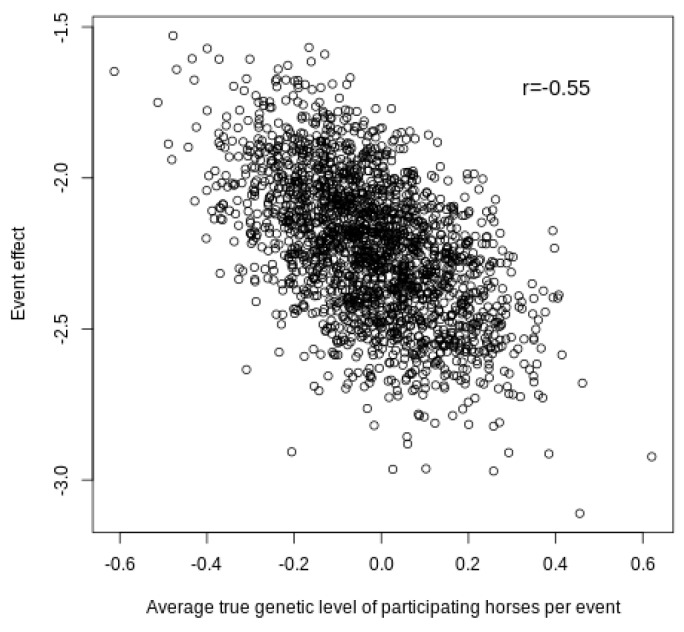
Two-dimensional plot of the estimate of the event effects and the average true genetic level for the liability of the ranking trait of the horses competing in an event.

The ability to capture the level of the competition events is illustrated by the results shown in Figure 4, which represent the estimates of the event effects and the average true additive genetic effect for the liabilities of the horses competing in each event.

As expected, there was a negative correlation (−0.55), which indicated that the most exigent events required a higher breeding value for the winner of the competition because the liability of the winner was set to zero. Therefore, competitions with the best horses (average genetic level) are associated with low “event” effects, while competitions of the worst horses have high “event” effects. In addition, Table 2 shows the predicted breeding values and the percentile within their ranking of the horses that competed in the highest and lowest estimated events in the simulation study.

In the event with the highest estimated effect, the predicted breeding values of 5 of 10 horses were ranked in the top 10%, and 8 out of 10 had a positive predicted breeding value. In contrast, in the event with the lowest estimated effect, all the horses had a negative predicted breeding value, and five had breeding values that were among the worst 10% of individuals. Nevertheless, the proposed approach might work well if the distribution of horses in events is random, as it was in the simulated example, or if the degree of structuring is small. In cases in which the structuring is high, Legarra and Ricard [10] proved with a simulation study that the results can be biased. Therefore, some other alternatives might be appropriate—e.g., the approach suggested by these authors, who proposed a mixture model in which horses are classified into several groups based on their performance. Further research is needed to adapt the GIBBSTHUR software for use in those scenarios.

## 6. Conclusions

The GIBBSTHUR software provides a useful tool for the breeding evaluation of ranking traits in horses. It can analyze ranking traits jointly with other categorical or continuous traits.

## Figures and Tables

**Table 1:** Phenotypic performance in the ranking trait of the horses with the highest and lowest predicted breeding value (EBV) for the liability of the ranking trait.

Place	Highest EBV	Lowest EBV
1	8	-
2	4	-
3	-	-
4	-	-
5	-	-
6	1	-
7	-	1
8	-	-
9	-	2
10	-	5

**Table 2:** Predicted breeding values and percentile within their ranking (between brackets) of the participants in the highest or lowest estimated events.

Position	Highest	Lowest
1st	1.55 (100.00)	−0.62 (9.64)
2nd	1.23 (99.50)	−0.69 (7.27)
3rd	0.69 (92.60)	−0.03 (48.19)
4th	0.86 (96.20)	−0.06 (46.19)
5th	0.73 (93.73)	−0.64 (9.03)
6th	0.46 (82.88)	−0.12 (40.58)
7th	0.03 (53.92)	−0.92 (2.65)
8th	0.32 (75.80)	−0.51 (13.73)
9th	−0.35 (22.00)	−0.31 (24.50)
10th	−0.15 (34.15)	−0.89 (3.07)
